# Incidence of conjunctivitis adverse event in patients treated with biologics for atopic dermatitis: A systematic review and meta-analysis

**DOI:** 10.1016/j.jdin.2023.05.014

**Published:** 2023-07-06

**Authors:** Rose Alraddadi, Abdulrahman H. Alsamadani, Mulham A. Kalantan, Yara E. Aljefri, Hadeel A. Maaddawi, Athoub N. Kadasa, Rahaf F. Alturkistani, Abdulhadi H. Jfri

**Affiliations:** a College of Medicine, King Saud Bin Abdulaziz University for Health Sciences, Jeddah, Saudi Arabia; b King Abdullah International Medical Research Center, Jeddah, Saudi Arabia; c Division of Dermatology, Department of Medicine, Ministry of National Guard Health Affairs, Jeddah, Saudi Arabia

**Keywords:** atopic dermatitis, biologics, dupilumab, IL-4/13, IL-13, lebrikizumab, tralokinumab

*To the Editor:* Atopic dermatitis (AD) is a chronic inflammatory skin disorder. Biologics that have been approved for AD include dupilumab (anti–interleukin (IL) 4/13) and recently a new anti–IL-13, namely tralokinumab, was included.^1^ Conjunctivitis was the most common adverse event from dupilumab from clinical trials and real-world experience.

Conjunctivitis is an inflammation or infection of the transparent membrane (conjunctiva) that lines the eyelid. It is caused by allergens, irritants, bacteria, and viral infections. Symptoms can vary depending on etiology, including redness, itching, burning, discharge, and eyelid edema.^2^ In dupilumab clinical trials, conjunctivitis is not observed for other indications, namely asthma and nasal polyps.^2^^,^^3^ We aimed to provide pooled incidence estimates using meta-analysis for the incidence of any conjunctivitis with dupilumab and other new agents, namely lebrikizumab and tralokinumab.

We systematically reviewed the randomized controlled trials pertaining to the incidence of any conjunctivitis subtype with dupilumab, lebrikizumab, and tralokinumab using preferred reporting items for systematic review and meta-analysis protocols (PROSPERO: 392077). A thorough search in Medline (PubMed), Directory of Open Access Journals, and ClinicalTrials.gov using the terms “Atopic dermatitis” and “biologics” or “Dupilumab” or “IL-4/13” or “Tralokinumab” or “IL-13” or “Lebrikizumab” (Fig 1). The search strategy is provided in Supplementary Materials (available via Mendeley at https://data.mendeley.com/datasets/xf7mm6x9m9/1).

**Fig 1:**
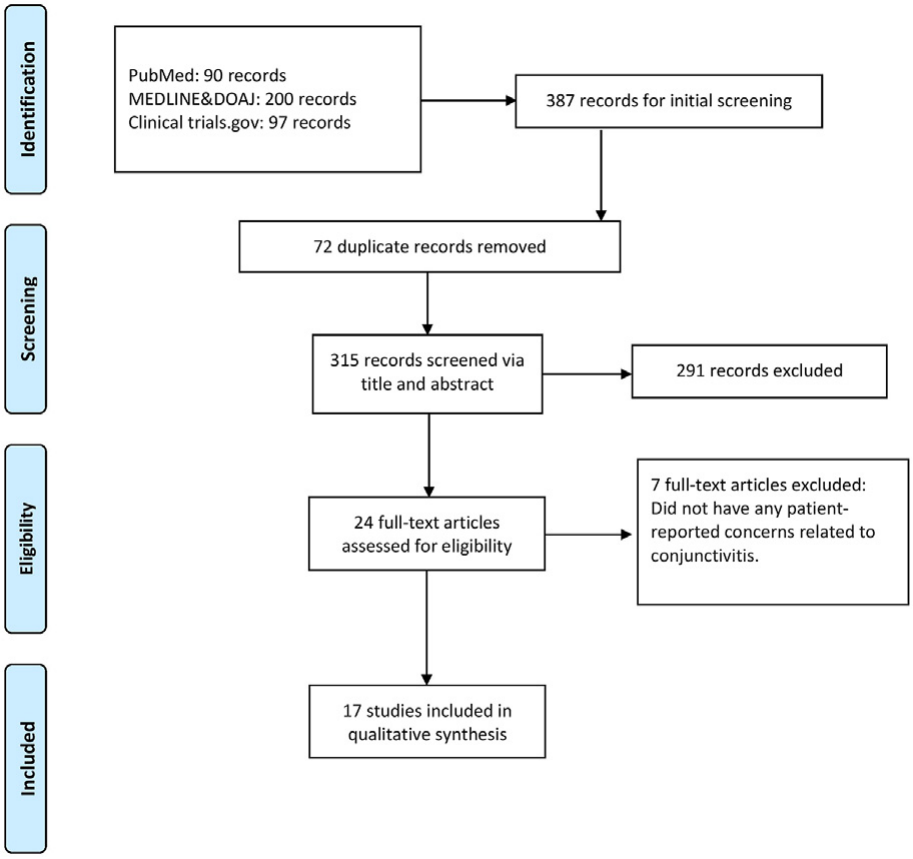
Study flowchart as per the preferred reporting items for systematic reviews and meta-analyses criteria.

Out of 263 titles and abstracts, a total of 17 studies with a cumulative sample size of 5830 participants reported the incidence of conjunctivitis among patients with AD undergoing biologic therapies. Out of 17 studies, 12 evaluated the efficacy of dupilumab, 2 tested lebrikizumab, and 3 assessed tralokinumab. Extent of heterogeneity across the studies was minimal (*Q* = 15.81, *I*^2^ = 0%).

Among 4197 patients undergoing biologic therapies, 213 reported conjunctivitis, whereas only 32 out of 1633 participants in the control group reported conjunctivitis. Pooled Mantel-Haenszel odds ratio was 3.11 (95% CI, 2.13-4.50) with no significant outlier effects (Fig 1). There was no publication bias (Egger’s regression *P* =.59) (Fig 2). No subgroup differences were found between different agents (*Q* = 0.23, *P* =.89).

**Fig 2:**
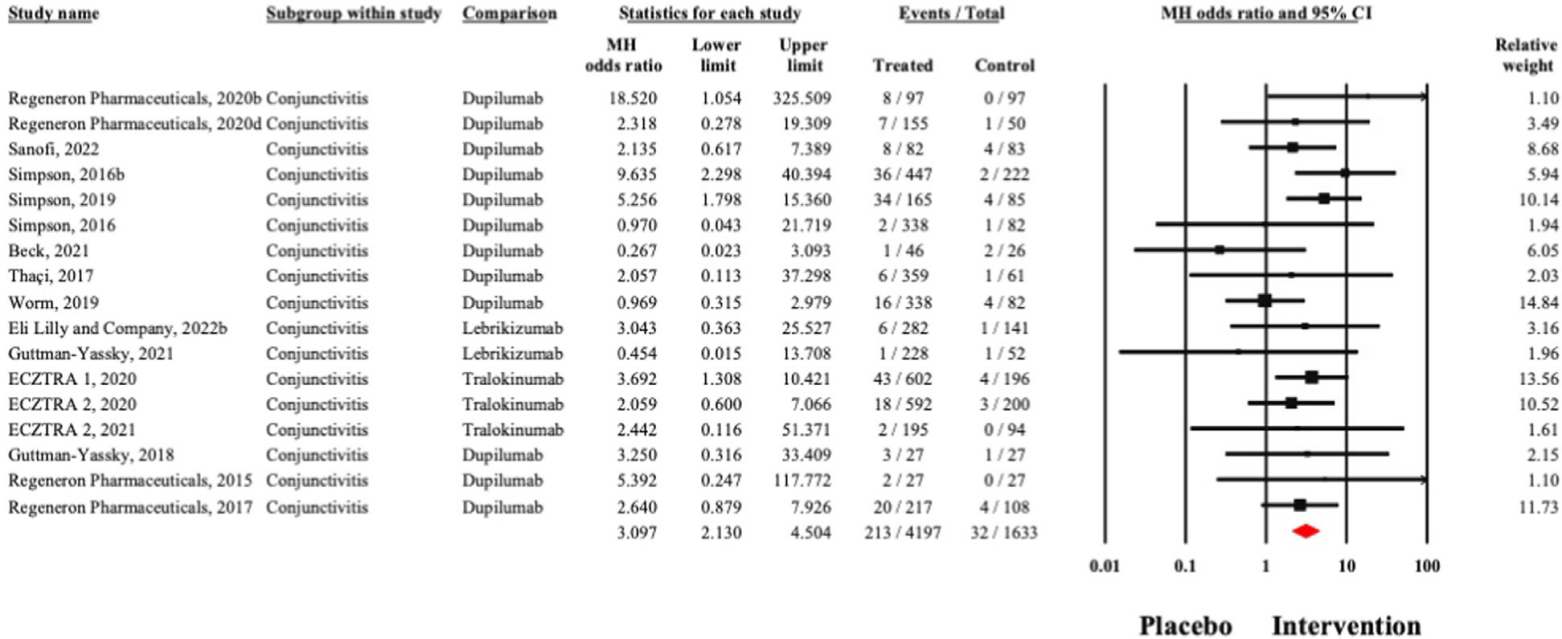
Forest plot presenting pooled effect size incidence of conjunctivitis adverse event with biologic therapies in atopic dermatitis.

The finding of this systematic review and meta-analysis demonstrated no statistical difference between the incidence of conjunctivitis between dupilumab and the included newly approved and in trial agents. Patients need to be counseled about the incidence of conjunctivitis and its occurrence with these new agents. The high incidence of conjunctivitis with the AD indication of these medications in contrast to the other indications of these drugs raise the possibility of an association related to AD itself. Sources of limitation could be the presence of minimal heterogeneity and the inability to portray causal relation between conjunctivitis and biologic agents.

## Conflicts of interest

None disclosed.
